# Supplementary material for: Surprise response as a probe for compressed memory states
Source: PLoS Comput Biol. 2020 Feb 3;16(2):e1007065. doi: 10.1371/journal.pcbi.1007065 (PMC7018098; doi:10.1371/journal.pcbi.1007065)
Supplement: S1 Table — The model parameters with the highest weighted-R2 for each of the subjects are presented for the NOC and for the IB models. For the IB model the N, β parameters were extracted from the individual maps shown in Fig 4c and in a similar manner N was extracted for the NOC model, as described in more detail in the Methods section. (PDF) [file pcbi.1007065.s009.pdf]

S1 Table

SubjectNo.	**N (NOC model)**	**N (IB model)**	β **(IB model)**
1	45	42	100
2	11	11	48.33
3	9	9	48.33
4	41	41	14.38
5	19	20	2.64
6	38	38	100
7	2	41	61.58
8	12	12	100
9	19	19	2.64
10	42	40	2.64
11	15	15	100
12	42	42	2.64
13	27	27	4.28
14	15	15	2.64
15	43	42	100
16	18	18	48.33
17	16	16	100

Model fit parameters for the NOC and IB models. The model parameters with the highest weighted-R^2 for each of the subjects are presented for the NOC and for the IB models. For the IB model the N, β parameters were extracted from the individual maps shown in fig. 4c and in a similar manner N was extracted for the NOC model, as described in more detail in the Methods section.
