# Supplementary material for: Surprise response as a probe for compressed memory states
Source: PLoS Comput Biol. 2020 Feb 3;16(2):e1007065. doi: 10.1371/journal.pcbi.1007065 (PMC7018098; doi:10.1371/journal.pcbi.1007065)
Supplement: S1 Text — (PDF) [file pcbi.1007065.s010.pdf]

Supporting information

for: **Surprise response as a probe for compressed memory states**
by Hadar Levi-Aharoni, Oren Shriki and Naftali Tishby

S1 Fig.

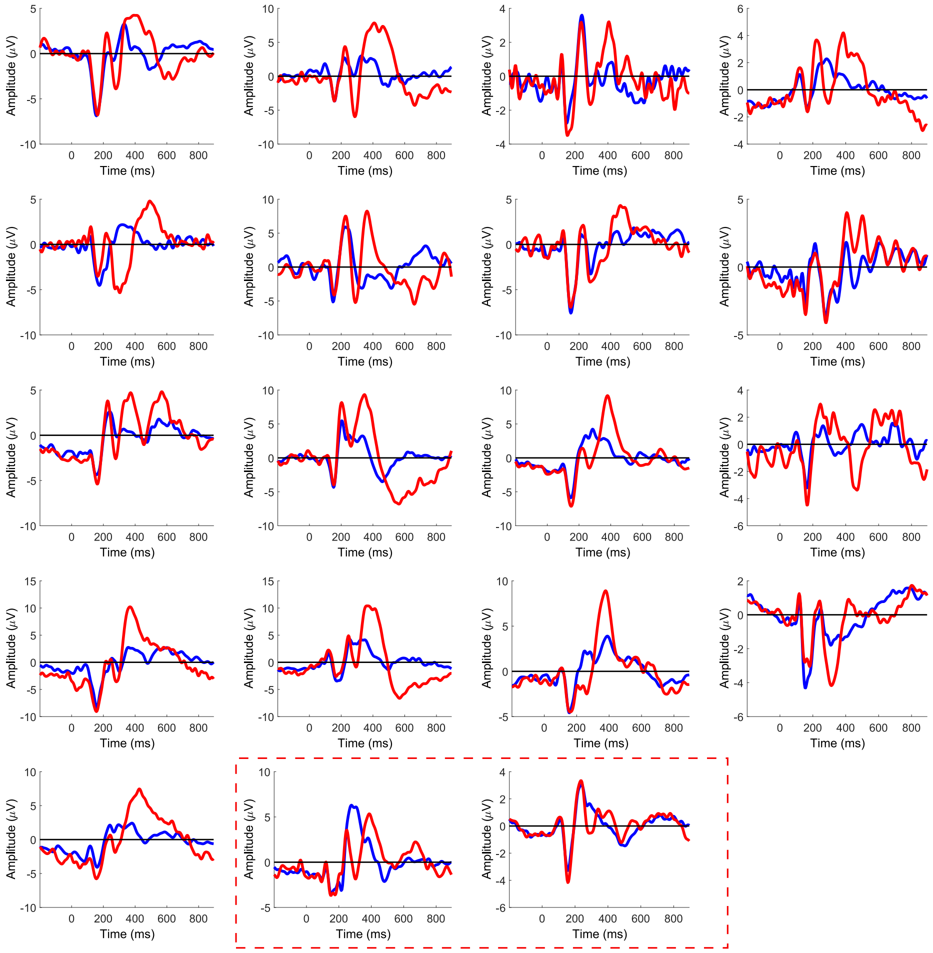

Single subject P300 ERPs. The P300 ERP of all subjects at electrode Cz is shown, each subject in a different plot. In red: the average of all oddball trials. In blue: the average of all standard trials. The last two plots marked by a dashed red rectangle correspond to two subjects where there was no clear difference between the oddball and standard curves and who therefore were omitted from the remainder of the analyses.

S2 Fig.

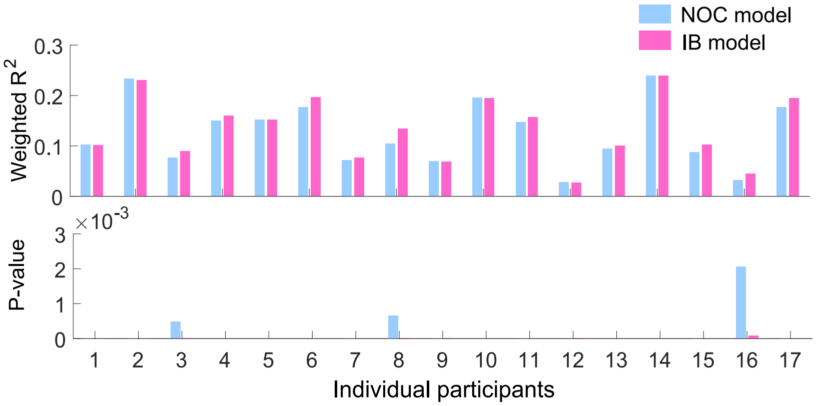

Subject-by-subject comparison of models performance. (**Top**) The weighted-R^2 of the two optimal models is compared for each subject. Each pair of blue (NOC model) and magenta (IB model) bars depict a different subject. (**Bottom**) The p-value for each model is shown for each participant. The p-value was calculated with a 1000-fold permutation test. The significance of $p - value = 0$ here is $p < 0.001$. For the details of the p-value calculation see Significance testing section in Methods.

S3 Fig.

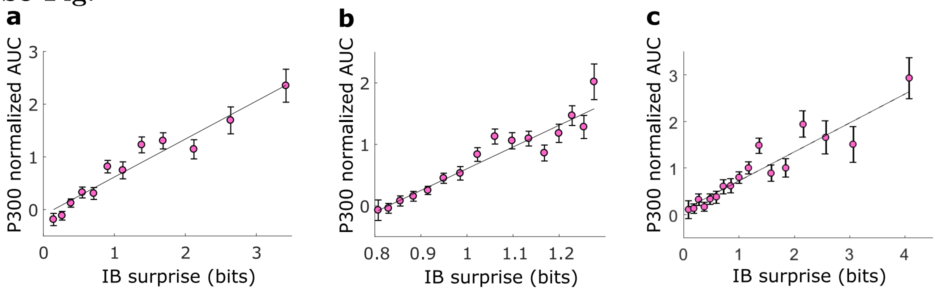

Single subject mean responses in the IB model. Average normalized P300 AUC responses as a function of the IB surprise for three different subjects. The fitted parameters for each subject were: (a) $N = 11$, $\beta = 48.33$ (b) $N = 15$, $\beta = 2.64$ (c) $N = 16$, $\beta = 100$. The weighted-R^2 values for the single-trials fit were: (a) 0.231 (b) 0.244 (c) 0.195. The R^2 values for the fit of the mean values (the plotted line) were: (a) 0.939 (b) 0.907 (c) 0.889. The error bars indicate the SEM.

S4 Fig.

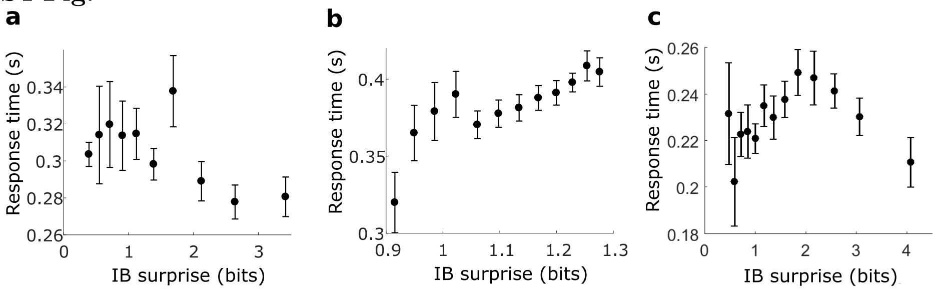

Single subject response time as a function of the IB surprise. Results for three subjects (same subjects as in supplementary S3 Fig), showing a non-linear and non-monotonous dependency of the RT on the IB surprise. This behavior was qualitatively different across subjects, showing that a multi-subject analysis is not straightforward for the RT and calls for a more complex model, presumably due to additional parameters affecting the response time. The surprise model parameters are as indicated in supplementary S3 Fig The error bars indicate the SEM.

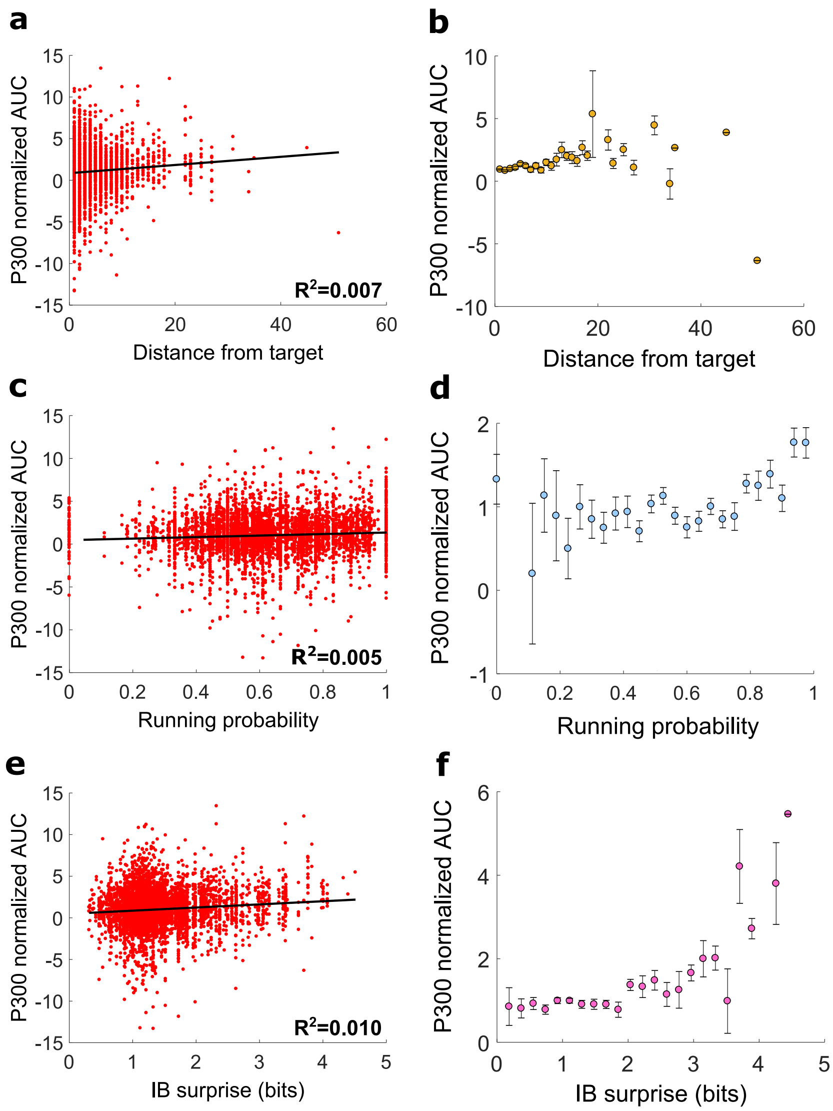

S5 Fig. A comparison of the NOC and IB models with the distance-from-target model on oddball trials. (a) Single-trial and (b) average normalized P300 AUC responses to oddball tones as a function of the distance (in number of elements) from the last oddball in the sequence, for all subjects. $R^2 = 0.007$, 5448 data points, error DOF = 5446, F-statistic vs. constant model: 37.3, p-value=1.06×10^{-09} (c) Single-trial and (d) average normalized P300 AUC responses to oddball tones as a function of the running probability, for all subjects. For each subject the best N was fitted as described in the main text. $R^2 = 0.005$, 5492 data points, error DOF = 5490, F-statistic vs. constant model: 29.3, p-value=6.39×10^{-08} (e) Single-trial and (f) average normalized P300 AUC responses to oddball tones as a function of the IB surprise, for all subjects. For each subject the best N and β were fitted as described in the main text. $R^2 = 0.01$, 5448 data points, error DOF = 5446, F-statistic vs. constant model: 55.3, p-value=1.21×10^{-013} The running probability and IB surprise were binned such that they have an identical number of values (28) on the x-axis as in (a). In all figures the error bars indicate SEM. Note that the scale of the y-axes on the right panels was accommodated to the relevant region, compared to the left panels which show the full range. Notice how the IB surprise shows a consistent increase in the AUC while explaining a large range of AUC responses.

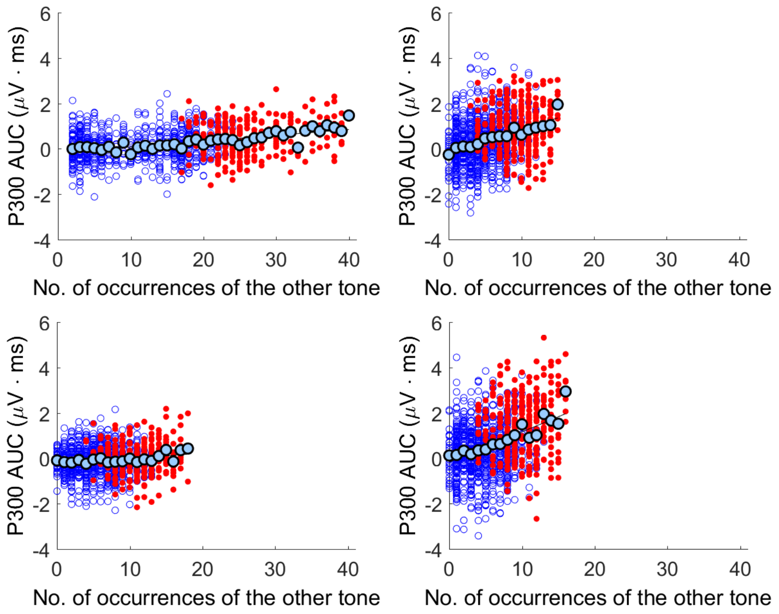

S6 Fig.

Single-trial analysis with the NOC model. Single-trial P300 AUC responses of four representative subjects to standard tones (blue empty circles) and to oddball tones (red, filled circles) as a function of the number of occurrences (n) of the opposite tone in the preceding sub-sequence of N tones (the fitted N for each subject, N=41,15,18,16 from left to right, top to bottom). Weighted-R^2=0.150,0.147,0.032,0.177. The black-edged filled circles show the average response for each n. The x- and y-axes were accommodated to show the same range in all subplots.

S7 Fig.

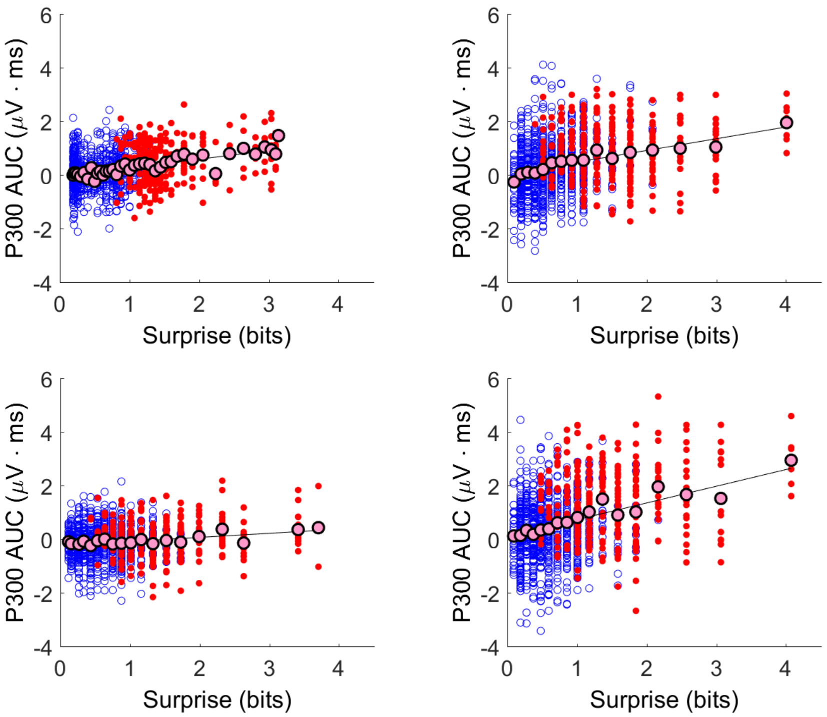

Single-trial analysis with the IB model. Single-trial P300 AUC responses to standard tones (blue, empty circles) and to oddball tones (solid red circles) as a function of the optimal IB surprise predictor (N=41,15,18,16 β=14.38,100,48.33,100 from left to right, top to bottom) for the same subjects as in S6 Fig. The black-edged solid circles show the average response for each surprise value. Weighted-R^2=0.160,0.157,0.045,0.195. The x- and y-axes were accommodated to show the same range in all subplots.

S1 Table

SubjectNo.	**N (NOC model)**	**N (IB model)**	β **(IB model)**
1	45	42	100
2	11	11	48.33
3	9	9	48.33
4	41	41	14.38
5	19	20	2.64
6	38	38	100
7	2	41	61.58
8	12	12	100
9	19	19	2.64
10	42	40	2.64
11	15	15	100
12	42	42	2.64
13	27	27	4.28
14	15	15	2.64
15	43	42	100
16	18	18	48.33
17	16	16	100

Model fit parameters for the NOC and IB models. The model parameters with the highest weighted-R^2 for each of the subjects are presented for the NOC and for the IB models. For the IB model the N, β parameters were extracted from the individual maps shown in fig. 4c and in a similar manner N was extracted for the NOC model, as described in more detail in the Methods section.

S8 Fig.

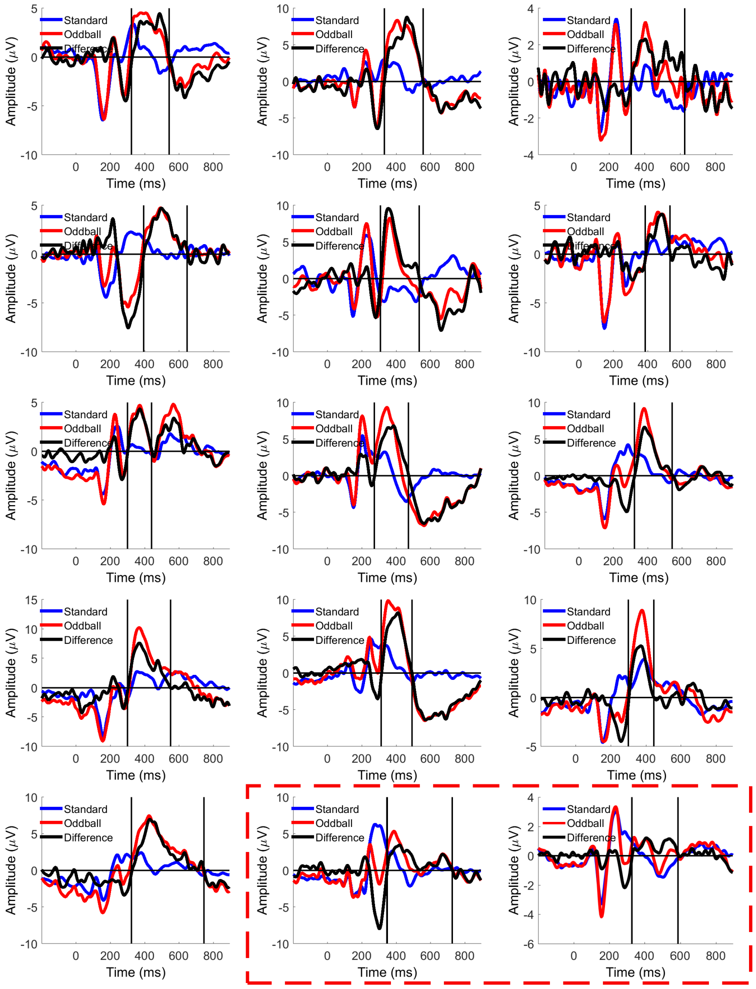

A subject-by-subject definition of the P300 area-under-the-curve (AUC) feature boundaries. The P300 ERPs are presented for all subjects with the definition of the P300 AUC (similarly as in Fig. 2b): event-related potentials averaged over all oddball (red) and standard (blue) trials in electrode Cz are shown for each subject. The difference between the oddball and standard curves (solid black) was used to determine for each subject the zero-crossing points (t_1 and t_2, solid vertical lines) around the P300 peak. The P300 AUC per trial was defined as the area between t_1 and t_2 on each trial. The last two plots marked by a dashed red rectangle correspond to the two subjects that were omitted from the remainder of the analyses (see the main text for more details).

S1 Text Alternative surprise models for the P300. In the context of the oddball paradigm, Tueting, Sutton and Zubin [1] showed in 1970 that the P300 amplitude is affected by the oddball probability of the sequence (among other factors [2]). This was followed by other studies described below which showed dependence of the P300 on the preceding sequence of tones, in addition to the effect of the a-priori oddball probability. In 1976 an innovative study by Squires et al. [3] suggested a model of trial-by-trial expectancy to account for fluctuations in the P300 amplitude due to an auditory oddball sequence. This was an impressive study, but the model had several components and only considered the influence of up to five preceding elements. A study a year later by Duncan-Johnson and Donchin [4] compared the effect of the a-priori probability relative to the effect of the preceding tone and found that both factors contributed to the P300 amplitude independently. However, this only characterizes very short term memory effects (one preceding tone).

More recently, a model by Mars et al. [5] considered infinitely long sequences in the past. The surprise of each event is modeled as the minus log of the probability associated with each event given all preceding trials. The probability is estimated using a maximum likelihood estimate and assuming a prior with equally likely events (formally assuming a uniform Dirichlet prior over the oddball probabilities). This was the first work, to the best of our knowledge, to give a formal account of the surprise in single trials and associate it with single trial amplitudes of the P300. What is missing from this work, in our view, is the eventuality of inter-subject differences in the surprise model (all subjects have the same model with infinite memory length).

Finally, Kolossa et al. [6] suggested a predictive surprise model based on digital filtering, combining both Mars' and Squires' models and redefining them as three additive digital filtering processes. The surprise is modeled as the minus log of the probability of the next element, where the probability is given as a sum of three components: a short-term memory contribution depending on the number of oddball occurrences in the entire sequence with a strong decaying memory factor, a long-term memory contribution with a slower decay factor, and an alternation term which depends on a few preceding elements. Kolossa's model parameters can be easily interpreted and connected to memory parameters; however, as Squires' model it seems to have a relatively large number of components and parameters.

Kolossa et al. thoroughly compared the above models [6] and also drew the attention to the difference between models of predictive surprise and models of Bayesian surprise. In models of predictive surprise the probability for the next element is estimated in each trial and the surprise in each trial is modeled as the minus log of this probability. Models of Bayesian surprise model the surprise as the revision in the internal probability distribution over the possible elements after each trial. This is the distance between the two estimated distributions, before and after observing each element. This can be quantified, for example, using the D_{KL} distance between the distributions. An example of a Bayesian surprise model was given by Ostwald et al. [7] for the somatosensory system under an oddball paradigm. However, Mars et al. [5] tested a Bayesian surprise model on their P300 data as an alternative model and found their predictive surprise model to give better results.

As we show in the main text, the dependency on the number of oddball occurrences is observed for a good theoretical reason: given a memory length, in the oddball paradigm the number of oddball occurrences in the preceding sequence is a minimal sufficient statistic [8] to predict the next tone. The models mentioned above are all dependent on this number, but Squires' and Kolossa's models contain more information about the exact sequence which is both unnecessary theoretically for efficient processing of the oddball sequence, and also do not seem to have a significant advantage in explaining the P300 data, as shown in Kolossa et al. Mars' model, on the other hand,

may lose important information by unifying all subjects in a single model.

It is also worth noting a related predictor for the P300 amplitude known as the target-to-target interval [9]; i.e., the number of non-target elements preceding the target element. This predictor considers only target trials and was used to analyze mean responses. A comparison with this model is shown in supplementary Fig. S5.

References

1. Tueting P, Sutton S, Zubin J. Quantitative Evoked Potential Correlates of the Probability of Events. Psychophysiology. 1970;7(3):385–394. doi:10.1111/j.1469-8986.1970.tb01763.x.

2. Polich J. Updating P300: an integrative theory of P3a and P3b. Clinical neurophysiology : official journal of the International Federation of Clinical Neurophysiology. 2007;118(10):2128–48. doi:10.1016/j.clinph.2007.04.019.

3. Squires KC, Wickens C, Squires NK, Donchin E. The Effect of Stimulus Sequence on the Waveform of Cortical Event-Related Potential. Science. 1976;193(6):92–94.

4. Duncan-Johnson CC, Donchin E. On quantifying Surprise: The Variation of Event-Related Potentials With Subjective Probability. Psychophysiology. 1977;14(5):456–467. doi:10.1111/j.1469-8986.1977.tb01312.x.

5. Mars RB, Debener S, Gladwin TE, Harrison LM, Haggard P, Rothwell JC, et al. Trial-by-Trial Fluctuations in the Event-Related Electroencephalogram Reflect Dynamic Changes in the Degree of Surprise. Journal of Neuroscience. 2008;28(47):12539–12545. doi:10.1523/JNEUROSCI.2925-08.2008.

6. Kolossa A, Fingscheidt T, Wessel K, Kopp B. A Model-Based Approach to Trial-By-Trial P300 Amplitude Fluctuations. Frontiers in Human Neuroscience. 2013;6(February):1–18. doi:10.3389/fnhum.2012.00359.

7. Ostwald D, Spitzer B, Guggenmos M, Schmidt TT, Kiebel SJ, Blankenburg F. Evidence for neural encoding of Bayesian surprise in human somatosensation. NeuroImage. 2012;62(1):177–188. doi:10.1016/j.neuroimage.2012.04.050.

8. Thomas M Cover JAT. Elements of Information Theory. 2nd ed. Wiley-Interscience; 2006.

9. Gonsalvez CJ, Polich J. P300 amplitude is determined by target-to-target interval. Psychophysiology. 2002;39(3):388–396. doi:10.1017/S0048577201393137.
